# Low‐ and high‐protein diets do not alter ex vivo insulin action in skeletal muscle

**DOI:** 10.14814/phy2.13798

**Published:** 2018-07-12

**Authors:** Zhencheng Li, Mette Line Rasmussen, Jingwen Li, Carlos Henríquez Olguín, Jonas Roland Knudsen, Ole Søgaard, Agnete B. Madsen, Thomas E. Jensen

**Affiliations:** ^1^ Section of Molecular Physiology Department of Nutrition, Exercise and Sports University of Copenhagen Copenhagen Denmark

**Keywords:** Dietary protein, insulin signaling, skeletal muscle

## Abstract

A low‐protein high carbohydrate (LPHC) diet and a high‐protein low carbohydrate (HPLC) diet have been reported to positively and negatively regulate whole‐body glucose tolerance and insulin sensitivity, respectively. Skeletal muscle is quantitatively the most important tissue clearing glucose in the postprandial state, but it is unclear if LPHC and HPLC diets directly influence insulin action in skeletal muscle. To test this, mice were placed on control chow diet, LPHC and HPLC diets for 13.5 weeks at which time the submaximal insulin‐stimulated glucose transport and insulin signaling were evaluated in ex vivo incubated oxidative soleus and glycolytic EDL muscle. At the whole‐body level, the diets had the anticipated effects, with LPHC diet improving glucose tolerance and insulin‐sensitivity whereas HPLC diet had the opposite effect. However, neither insulin‐stimulated Akt/TBC1D4 signaling and glucose transport ex vivo, nor cell signaling in vivo were altered by the diets. These data imply that skeletal muscle insulin sensitivity does not contribute to the whole‐body effects of LPHC and HPLC diets on glucose metabolism.

## Introduction

The relative dietary protein content is increasingly recognized to markedly influence whole‐body insulin sensitivity. Thus, lowering the protein content to <~10 energy (E) % is associated with increased whole‐body glucose tolerance and increased insulin sensitivity measured as HOMA‐IR index in mice and humans (Laeger et al. 2014; Solon‐Biet et al. 2014, 2015; Maida et al. 2016). Conversely, diets deriving a high percentage of total energy from protein, negatively affect whole‐body glucose metabolism in mice (Solon‐Biet et al. 2014, 2015).

Mechanistically, release of the stress hormone FGF21 from the liver is a hallmark response to LPHC diets and FGF21 is required for many of the beneficial effects of low‐protein diets, including the effects on whole‐body insulin‐sensitivity (Laeger et al. 2014; Fontana et al. 2016; Maida et al. 2016). The LPHC diet‐stimulated rise in FGF21 has been shown to increase adiponectin, a hormone proposed to mediate the effects of FGF21 on insulin sensitivity in different organs, including skeletal muscle (Lin et al. 2013; Vu et al. 2013; Maida et al. 2016; Ahlstrom et al. 2017; Li et al. 2018). Adiponectin stimulation has been shown to activate AMPK in skeletal muscle (Zhou et al. 2009; Yoon et al. 2006; Iwabu et al. 2010), a protein necessary and sufficient to increase skeletal muscle insulin sensitivity via a muscle‐intrinsic mechanism, that is, retained in isolated incubated muscles (Kjobsted et al. 2015, 2017). In addition, LPHC diet and the resulting increases in FGF21 and adiponectin might inhibit several factors suggested to cause insulin resistance in the diet‐induced obese setting, including ceramides, ectopic lipid accumulation, and pro‐inflammatory skewing of macrophages (Holland et al. 2013; Laeger et al. 2014, 2016; Maida et al. 2016; Li et al. 2018). Decreasing these factors might also influence insulin action in the isolated muscle.

In relation to increased protein intake, branched chain amino acids (BCAAs) have repeatedly been associated with insulin resistance in rodents and humans (Newgard et al. 2009; Solon‐Biet et al. 2014). Importantly, specific lowering of BCAAs in an otherwise isocaloric and isonitrous diet, or a high fat‐high sugar western diet, improved insulin resistance in obese insulin‐resistant rodent models (White et al. 2016; Cummings et al. 2018). BCAAs were proposed to cause rodent skeletal muscle insulin resistance by several mechanisms: (1) activation of mTORC1 in skeletal muscle to impair insulin signaling at the level of IRS1 (Newgard et al. 2009; Lynch and Adams 2014). (2) increased substrate load on mitochondria to impair lipid oxidation and promote accumulation of incompletely oxidized fatty acids (Koves et al. 2008; Lerin et al. 2016; White et al. 2016) and (3) stimulation of transendothelial fatty acid transport to promote skeletal muscle lipotoxicity (Jang et al. 2016). The latter in particular might negatively influence skeletal muscle insulin action in a muscle‐intrinsic manner ex vivo similar to diet‐induced obese models (Frosig et al. 2013; Sylow et al. 2014).

Skeletal muscle is quantitatively the largest contributor to whole‐body glucose disposal during insulin‐stimulated conditions (DeFronzo et al. 1981), and reduced skeletal muscle insulin action is also an early contributor and determinant of systemic glucose intolerance in the pathophysiology of type 2 diabetes (DeFronzo and Tripathy 2009). Therefore, we undertook the current study to directly test if the whole‐body effects of a 5E% protein LPHC or a 40E% HPLC diet provided for 13.5 weeks would elicit muscle‐intrinsic changes in insulin‐sensitivity. However, despite confirming the previously reported beneficial and detrimental effects of LPHC and HPLC diets on whole‐body glucose metabolism, respectively, insulin action in isolated incubated skeletal muscle was unaffected. This suggests that the effects of low/high protein diets on glucose metabolism and insulin sensitivity are independent of skeletal muscle insulin signaling and glucose transport regulation.

## Methods

### Experimental overview

**Figure 1:**
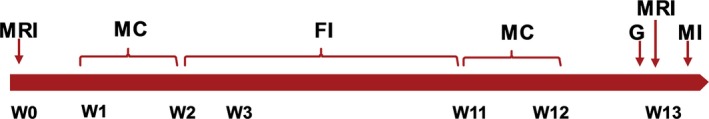
Schematic overview of the experimental design. BC, body composition. FI, food intake; MC, metabolic cage; G, glucose tolerance test; MI, muscle incubation and tissue harvest; W, week.

Chow, LPHC and HPLC diets were purchased from Speciality Feeds (Australia) and are described in Table 1. Following acclimation for 1 week, 8–9 months old C57BL/6JRj female retired breeder mice (Janvier Labs, France) were randomized to Chow (*n* = 7, one mouse died during the experiment), LPHC (*n* = 8) or HPLC (*n* = 8) groups and were group‐housed at 22–24°C on a 12‐h light/12‐h dark‐cycle with ad libitum access to water and diets for 13.5 weeks (Table 1 and Fig. 1). Retired female breeders were chosen to validate the diet effects in middle‐aged mice for a separate follow‐up study and because the thin muscles from female mice are in theory less prone to hypoxia during ex vivo incubation (Bonen et al. 1994). Magnetic resonance imaging was performed at week (W) 0 and W13 to determine body composition. Food intake was measured weekly from W2 to W11. In W11, the mice were single‐housed on their respective diets for 3 days before performing indirect calorimetry and activity measurements for 24 h. 1 day before the end of W13, a modified glucose tolerance test (GTT) was performed. The mice were then allowed to recover for 4 days before the terminal experiment with harvest of tissues and ex vivo muscle incubation. The individual procedures and analyses are detailed below.

**Table 1:** Dietary composition

		Chow	LPHC	HPLC
Net metabolizable energy (MJ/Kg)		14.4	15.6	15.5
Total calculated netmetabolizable energy from (%)	Lipid	17.8	20.7	30.5
	Protein	18.8	4.8	40.6
	Carbohydrate	63.4	74.5	28.9
Calculated amino acids (%)	Valine	1.26	0.36	3.05
	Leucine	1.8	0.5	4.2
	Isoleucine	0.87	0.29	2.45
	Threonine	0.79	0.21	1.8
	Methionine	0.84	0.23	1.93
	Cysteine	0.05	0.04	0.36
	Lysine	1.49	0.38	3.19
	Phenylalanine	0.99	0.27	2.31
	Tyrosine	1.04	0.31	2.59
	Tryptophan	0.27	0.07	0.6
	Histidine	0.6	0.16	1.39
Ingredient (g/kg)	Casein	200	55	462
	Sucrose	100	66	67
	Soy oil	70	88	129
	Cellulose	50	0	52
	Wheat starch	404	650	143
	Dextrinised starch	132	99	100
	DL methionine	3	0.8	6.9
	Calcium carbonate	13.1	13.1	13.1
	Sodium Chloride	2.6	2.6	2.6
	AIN93 trace minerals	1.4	1.4	1.4
	Potassium dihydrogen phosphate	6.9	6.9	6.9
	Potassium sulfate	1.6	1.6	1.6
	Potassium citrate	2.5	2.5	2.5
	Choline Chloride (75%)	2.5	2.5	2.5
	AIN93 vitamins	10	10	10
Physical property	Pellet diameter (mm)	12	12	12
	Color	White	White	White
	Cereal/semi. pure	Semi. pure	Semi. pure	Semi. pure
Reference number		SF14‐162	SF09‐048	SF09‐069

LPHC, low protein high carbohydrate diet; HPLC, high protein low carbohydrate diet.

### Body composition (BC) determination

After weighing, BC of individual conscious fed mice was determined in an EchoMRI™ 4‐in‐1‐500 Body Composition Analyzer between 9 and 12 am according to the manufacturer′s instructions.

### Indirect calorimetry and habitual activity

Three days before the measurement, mice were acclimated to single housing in metabolic cages (PhenoMaster, TSE, Germany) with free access to food and water gel. After acclimation, habitual activity, measured as laser beam break counts, expired carbon dioxide (CO_2_), and oxygen (O_2_) consumption were monitored over the next 2 days. The data from the last 24 h were used in the analysis.

### Energy intake

Food intake was measured three times per week between 9 and 11 am. In brief, the weight of the food pellets was measured before and after placement on the cage. The food intake was converted into energy intake by multiplying with the net metabolizable energy and then divided by the total number of mice in a given cage.

### Modified glucose tolerance test (GTT) and plasma collection

On the experimental day, the mice were weighed, single‐housed in clean cages and deprived of food from 7 to 8 am for 6 h. A glucose solution (1 g/5 mL) was prepared by dissolving glucose in saline 1 h before the GTT. Blood glucose was monitored, using a standard glucometer (Contour XT, Bayer's). For plasma isolation and insulin measurements, ~50 *μ*L of blood was collected pre and at 30 min postintraperitoneal injection of the glucose solution (10 *μ*L/g body weight). After the last blood samples were drawn, the mice were immediately allowed access to their respective diets again.

### Tissue harvest and muscle incubation

Mice were fasted for 2–3 h from 9 am. Extensor digitorum longus (EDL) and soleus (SOL) muscles were carefully dissected from mice anesthetized with pentobarbital 6 mg and 0.2 mg lidocaine/100 g body weight. The excised muscles were pinned vertically at approximately resting length onto a custom‐made silicon‐base on plastic holders and placed in glass tubes containing Krebs–Ringer–Henseleit (KRH) buffer supplemented with 2 mmol/L pyruvate and 8 mmol/L mannitol in glass tubes with continuous bubbling with 95% O_2_ and 5% CO_2_ at 30°C. After 30 min, the plastic holders with muscles were transferred to new tubes containing KRH ± 1.8 nmol/L insulin to elicit a submaximal stimulation of insulin signaling and glucose transport (Frosig et al. 2013). After 10 min insulin stimulation, the plastic holders were transferred to new tubes containing KRH ± 1.8 nmol/L insulin in tracer medium (1 mmol/L 2‐deoxy glucose (2DG), 2‐[2,6‐^3^H] deoxy‐D‐glucose and [1‐^14^C] mannitol (Amersham Biosciences; specific activities of the two tracers in the medium were 0.13 and 0.11 *μ*Ci mL^−1^, respectively) for 10 min. Finally, the muscles were quickly rinsed in ice‐cold KRH, blot‐dried on paper tissue and snap frozen in liquid N_2_.

Tibialis anterior (TA) muscles were collected and snap frozen in liquid N_2_ before excision of EDL muscles. After EDL and SOL were dissected out, a piece of the most ventral lobe of the liver was collected and snap frozen in liquid N_2_. Blood was then collected from the punctured heart and thoracic cavity and centrifuged to obtain plasma.

### Plasma FGF21, insulin measurements and HOMA2‐IR

Plasma FGF21 and insulin concentration were measured, using ELISA kits from BioVendor (Cat. RD291108200R) and ALPCO (Cat. 80‐INSMSU‐E10), respectively. The measurements were performed strictly according to the manufacturers' instructions. HOMA2‐IR, a validated estimate of insulin resistance in mice (Mather 2009), was calculated using the fasting glucose and insulin values.

### Liver and muscle glycogen content

However, 10–30 mg of frozen liver tissue or TA muscle and 200 *μ*L of 1 N HCL were added to 1.5 mL tubes and boiled on a heating block at 98°C for 2 h. Glycogen content was then measured as glycosyl units after acid hydrolysis and glucose was determined fluorometrically from the neutralized perchloric acid extracts (Lowry and Passonneau 1972).

### Protein extraction

Here, 15 mg of liver or TA muscle, or the entire EDL and SOL muscles, trimmed free of visible fat and tendons, and weighed, were added to ice‐cold 2 mL centrifuge tubes containing a steel bead and 300 *μ*L lysis buffer (0.05 mol/L Tris Base pH 7.4, 0.15 mol/L NaCl, 1 mmol/L EDTA and EGTA, 0.05 mol/L sodium flouride, 5 mmol/L sodium pyrophosphate, 2 mmol/L sodium orthovanadate, 1 mmol/L benzamidine, 0.5% protease inhibitor cocktail (P8340, Sigma Aldrich), and 1% NP‐40) and lysed for 1 min at 30 Hz on a shaking bead‐mill (TissueLyser II, Qiagen, Valencia, CA, USA). The samples were then rotated end‐over‐end at 4°C for 30 min followed by centrifugation at 18.320*g* at 4°C for 20 min to recover supernatants (lysates) for western blotting and 2DG transport determination.

### Lysate preparation for Western blotting

The protein content of liver or muscle lysate was determined, using the BCA method (ThermoFisher, Cat. 23235). Thereafter, equivalent amounts of protein were mixed with 6× Laemmli sample buffer (11% SDS, 225 mmol/L Dithiothreitol (DTT), 340 mmol/L Tris, pH 6.8, 0.05% bromophenol blue, 20% glycerol) and appropriate amounts of deionized water and boiled at 95°C for 5 min.

### Western blotting

Equivalent amounts of protein from liver, TA, EDL or SOL were subjected to 5–15% SDS‐PAGE and semi‐dry transferred to PVDF membranes. After that, the membranes were blocked in 3–5% BSA or skim milk, depending on the primary antibody to be used at room temperature for 1 h followed by overnight incubation with primary antibody. The next day, the membranes were washed with TBS‐T and incubated with the relevant horseradish peroxidase‐conjugated secondary antibodies for 1 h and washed with TBS‐T again. The amount of bound antibody was measured using enhanced chemiluminescence (ECL+; Amersham Biosciences, Little Chalfont, UK) and images for densitometry were captured, using a ChemiDoc MP Imaging System (Bio‐Rad, Herules, CA, USA). After development, the membranes were stained with Coomassie Brilliant Blue to verify even transfer and similar total protein loading.

For the cell culture positive control lysates, confluent 6‐well dishes with fed HEK293 cells were lysed in 100 *μ*L of lysis buffer, and HeLa cells lysates stimulated with 100 nmol/L calyculin A for 30 min was a gift from Abcam. Both lysates were then further processed as above.

The primary antibodies used were phospho‐eIF2*α* Ser51 (Cell Signaling Technology (CST), 9721), phospho‐GCN2 Thr899 (Abcam, ab75836), phospho‐Erk1/2 Thr202/Tyr204 (CST, 4370), phospho‐p38 MAPK Thr180/Tyr182 (CST, 9211), phospho‐AMPK Thr172 (CST, 50081), phospho‐Akt Thr308 (CST, 13038), phospho‐Akt S473 (CST, 4060), phospho‐4E‐BP1 Ser65 (CST, 9451), phospho‐S6 Ser235/236 (CST, 4858), phospho‐p70S6K Thr389 (CST, 9206), phospho‐TBC1D4 Thr642 (CST, 8881), phospho‐TBC1D1 S237 (Millipore, 07‐2268), phospho‐ACC S212 (Millipore, 03 303), Perilipin2 (Novus Biologicals, NB110‐40877), Akt2 (CST, 2964) and p70S6K (CST, 9202), Hexokinase II (HKII) (CST, 2867), GLUT4 (ThermoFisher Scientific, PA‐23052).

### 2‐Deoxy‐d‐glucose (2DG) transport

For analysis of 2DG transport, 100 of 300 *μ*L total muscle lysate were mixed with 2 mL of Ultima Gold™ scintillation cocktail (Perkin Elmer, MA, USA). Then the samples were vigorously shaken for 3 min and radioactivity was measured using liquid scintillation counting (Tri‐Carb 2000; Packard Instrument, Downers Grove, IL).

### Statistical analyses of data

Results are expressed as means ± SEM. Statistical tests were performed using 2‐way ANOVA or repeated measurements ANOVA followed by Tukey′s post hoc test where appropriate using SPSS 22 and GraphPad Prism 7. In cases where the data could not pass Levene's equal variance test even after transformation, a nonparametric test was applied as indicated in the figure legend. The significance level was set at *P* < 0.05. Number written above bars in figures highlights a tendency to significant *P* value of *P* < 0.1.

## Results

**Figure 2:**
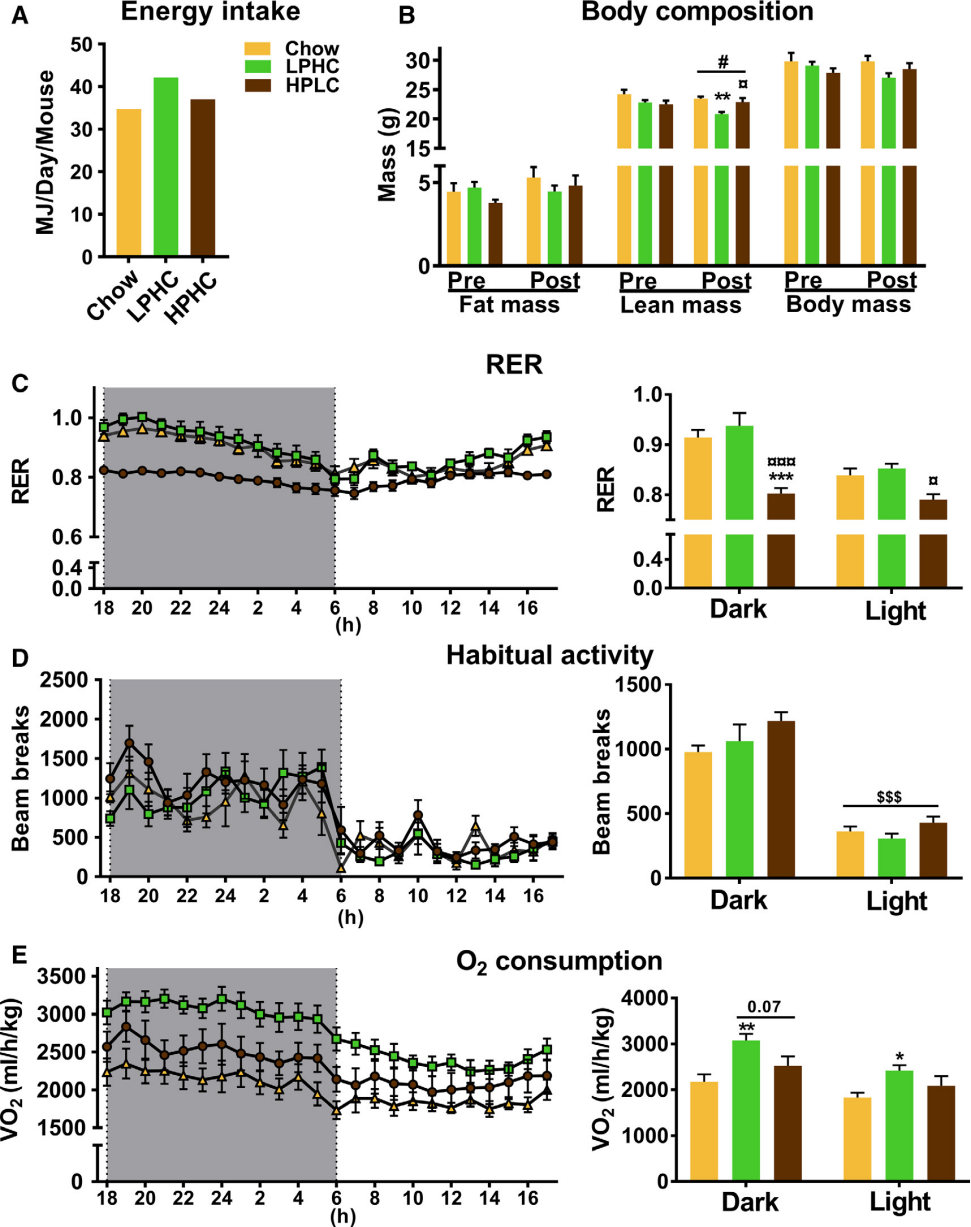
Chronic low protein high carbohydrate diet (LPHC) increased metabolic rate. (A) Average energy intake calculated as whole cage energy consumption/number of mice in a given cage. The color legend in panel A is shared with panel B–E. (B) Fat mass, lean mass and body mass measured pre‐ and post‐12 weeks of dietary intervention. #*P* < 0.05 main effect of dietary intervention. **P < 0.01 versus chow/*P* < 0.05 versus LPHC within lean post. (C–E) Respiratory exchange ratio (RER), habitual activity and oxygen consumption, respectively, in week 12 of the dietary intervention were recorded for 24 h, from which the average RER, habitual activity and oxygen consumption within light and dark‐phase were calculated (C, D & E right). For (C), interaction effect (*P* = 0.027); ***P < 0.001 versus chow/*P* < 0.001 versus LPHC within dark; *P* < 0.05 versus LPHC within the light phase. For (D), $$$*P* < 0.001 main effect of light phase. For (E), */**P < 0.05/0.01 versus chow within dark phase; Kruskal–Wallis test, a nonparametric test, followed by Dunn's multiple comparison test was applied to evaluate significance within light phase. Data are expressed as mean ± SEM. *N* = 7 for chow and *n* = 8 for LPHC/HPLC.

To test the effects of dietary protein content on skeletal muscle insulin sensitivity, 8–9 months old middle‐aged female C57BL/6 mice were placed in groups of 7–8 mice on either chow diet deriving 64/19/18E% from carbohydrate/protein/fat, LPHC diet deriving 75/5/21E% from carbohydrate/protein/fat or HPLC diet deriving 29/41/31E% from carbohydrate/protein/fats (Table 1). These diets were selected based on their efficacy in a previous study (Solon‐Biet et al. 2014). As expected, mice on the LPHC diet consumed more energy on average than chow‐fed mice (Fig. 2A). There were no changes in body or fat mass on these diets, but mice on the LPHC diet had a slight decrease in lean body mass in W13 compared to the other groups (Fig. 2B). In the respiratory chamber, measurements performed in W11 of the diet‐study, respiratory exchange ratio (RER) was lower in mice on HPLC diet (Fig. 2C), habitual activity was not different (Fig. 2D) and the O_2_ consumption was stimulated by the LPHC diet (Fig. 2E). Collectively, these data are well in line with previous data showing that LPHC diet induces hyperphagia and O_2_ consumption independent of habitual activity, likely by stimulating adaptive thermogenesis in brown and white adipose tissues.

**Figure 3:**
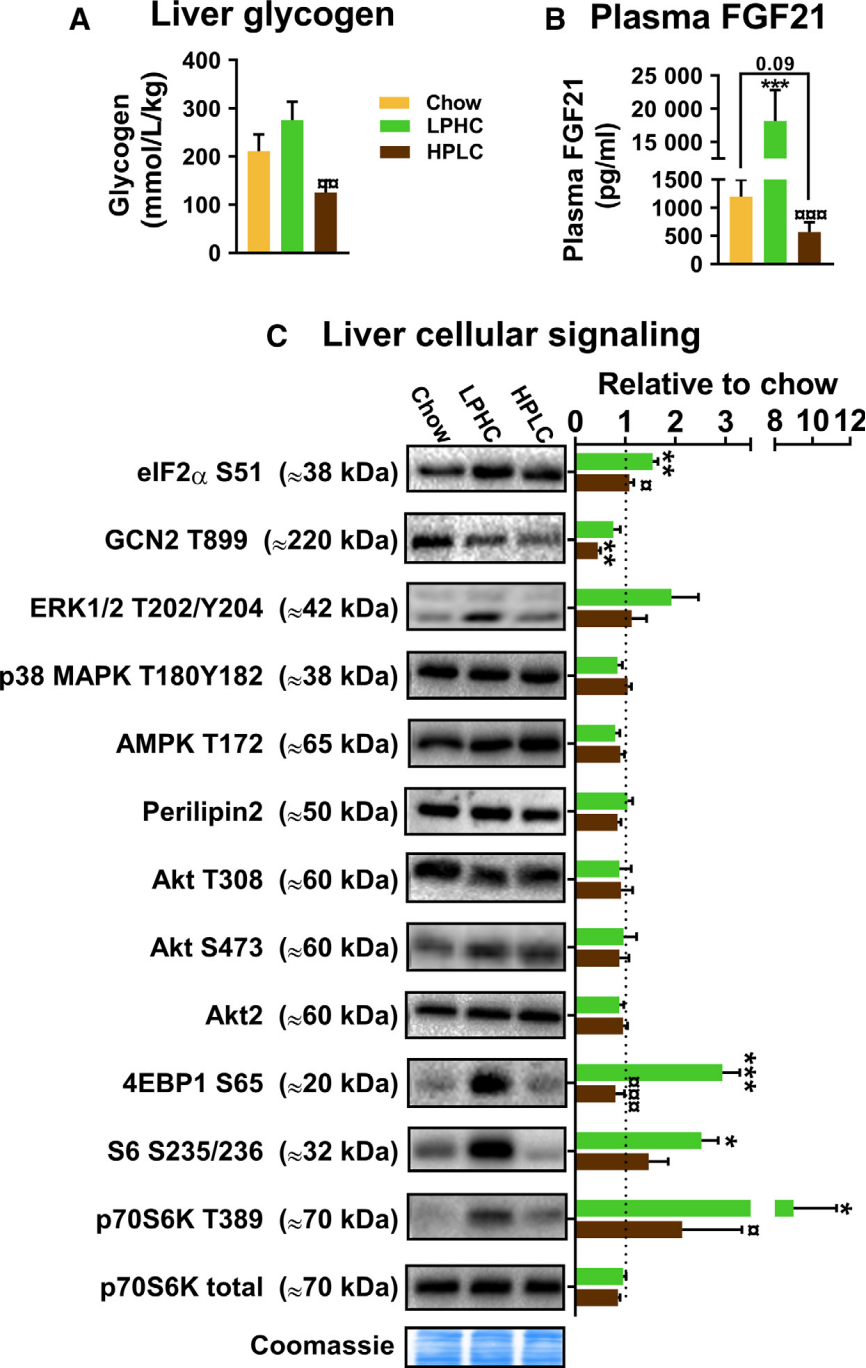
Chronic low protein high carbohydrate diet (LPHC) increased plasma FGF21 and altered liver cellular signaling. (A) Liver glycogen content. The color legend in panel A is shared with panel B–C. *P* < 0.01 versus LPHC. (B) Plasma FGF21. ***P < 0.001 versus chow/*P* < 0.001 versus LPHC. (C) Liver cellular signaling determined by western blotting. The signal intensities are presented relative to chow (dashed line). Representative blots and a coomassie loading control are shown to the left. Kruskal–Wallis test, a nonparametric test, followed by Dunn's multiple comparison test was applied to evaluate significance within S6 S235/236. HPLC, high protein low carbohydrate diet. */**/***P < 0.05/0.01/0.001 versus chow/ *P* < 0.05/0.001 versus LPHC. Data are expressed as mean ± SEM. *N* = 7 for chow and *n* = 8 for LPHC/HPLC.

Liver plays a key role in the adaptive response to LPHC diet by releasing the stress hormone FGF21, which is regulated by both protein and carbohydrate content of the diet (Solon‐Biet et al. 2016). As expected, the carbohydrate content of the diets was reflected in the liver glycogen (Fig. 3A). Also, the LPHC diet dramatically increased plasma FGF21 compared to chow (Fig. 3B), while the HPLC diet group showed a tendency (*P* = 0.09) toward lower FGF21. FGF21 release from liver is described to involve a GCN2/eIF2*α*‐dependent signaling pathway. Presently, the LPHC but not the HPLC diet increased eIF2*α* Ser51phosphorylation but not GCN2 Thr899 autophosphorylation (Fig. 3C). In contrast, mTORC1 substrate phosphorylation was surprisingly increased much more potently by the LPHC diet than by the HPLC diet (Fig. 3C), differing markedly from earlier reports (Solon‐Biet et al. 2014; Maida et al. 2017). However, we did not further investigate the reason for this discrepancy since this was not the aim of the study. No other measured phosphorylations or proteins responded significantly to the diets, including phosphorylation of ERK, p38 MAPK, AMPK, Akt, and expression of Perilipin 2 and Akt2 (Fig. 3C).

**Figure 4:**
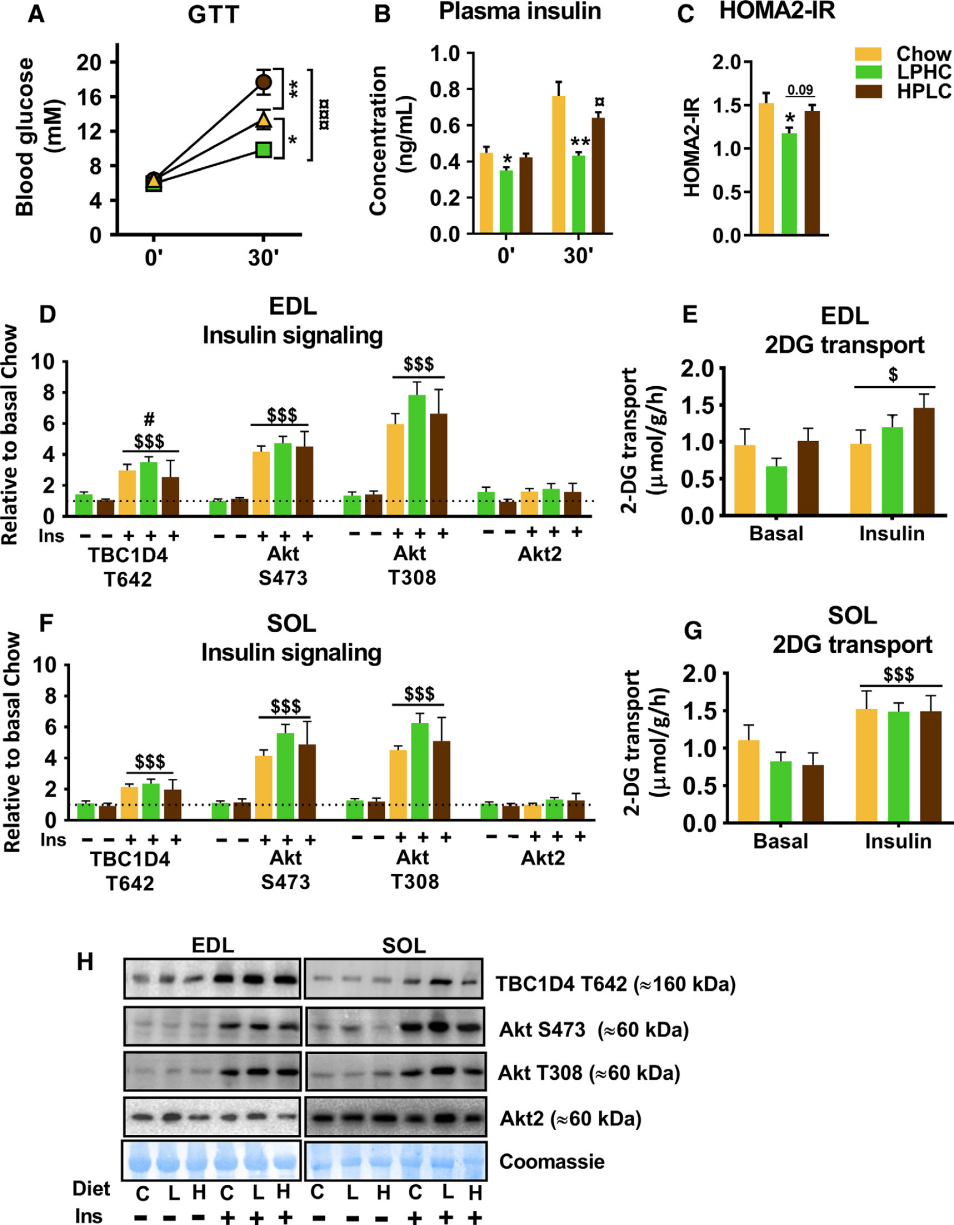
Alterations in whole body glucose tolerance by low protein high carbohydrate diet (LPHC) and high protein low carbohydrate diet (HPLC) are not reflected in isolated skeletal muscles. (A) A 30 min intra‐peritoneal glucose challenge (GTT) was performed one day before the end of week 13, during which blood samples were drawn before and at 30′ to measure the corresponding plasma insulin and to calculate HOMA2‐IR in panel B and C, respectively). The color legend in panel C is shared with panel A–G. For (A), interaction effect (*P* < 0.001); */**P < 0.05/0.01 versus chow/*P* < 0.01 versus LPHC within 30′. For (B), Kruskal–Wallis test, a nonparametric test, followed by Dunn's multiple comparison test was applied to evaluate significance within 30′; *P < 0.05 versus chow within 0′; **P < 0.01 versus chow/*P* < 0.05 versus LPHC within 30′. For (C), *P < 0.05 versus chow. For (D–G), Submaximal insulin‐stimulated muscle signaling and corresponding ex vivo 2‐Deoxy‐D‐glucose (2DG) transport measured in D & E) for Extensor digitorum longus (EDL), and in F &G) for soleus (SOL) muscles. For D–G, #*P* < 0.05 main effect of dietary intervention; $/$$$*P *< 0.05/0.001 main effect of insulin. (H) Representative blots and coomassie loading control for E & F. C, chow; L, LPHC; H, HPLC. Ins, insulin. Data are expressed as mean ± SEM. *N* = 7 for chow and *n* = 8 for LPHC/HPLC in A, B, E and F. One EDL was lost during processing, therefore, the basal HPLC groups in C + D were *n* = 7.

To evaluate the impact of diet on whole‐body insulin sensitivity, a modified glucose tolerance test was performed. Blood glucose and plasma insulin were measured before and 30 min after injection of a standardized glucose load. In this setup, glucose tolerance was augmented by the LPHC diet and impaired by the HPLC diet compared to chow (Fig. 4A). This was associated with a change in whole‐body insulin sensitivity since both the fasting and glucose‐stimulated insulin concentration was lower in the LPHC group compared to the two other groups, whereas the insulin levels on the HPLC diet were comparable to chow (Fig. 4B). Thus, our diets recapitulate previous findings of increased and decreased whole‐body glucose tolerance and insulin sensitivity in the context of LPHC and HPLC diet, respectively.

After 13.5 weeks on chow, LPHC or HPLC diet, we performed ex vivo incubations to evaluate possible muscle‐intrinsic changes in insulin sensitivity. To achieve this, we measured cell signaling and glucose transport in the slow‐twitch oxidative soleus muscle and the fast‐twitch glycolytic EDL muscle at rest or during stimulation with a submaximal 1.8 nmol/L insulin, that is, an insulin concentration in which detection of either increased or decreased insulin sensitivity should be possible. In both muscles, insulin had significant stimulatory effects on Akt and TBC1D4 phosphorylation (Fig. 4C and E) and glucose transport (Fig. 4D and F). However, there were no discernible effects of diet on these parameters.

**Figure 5:**
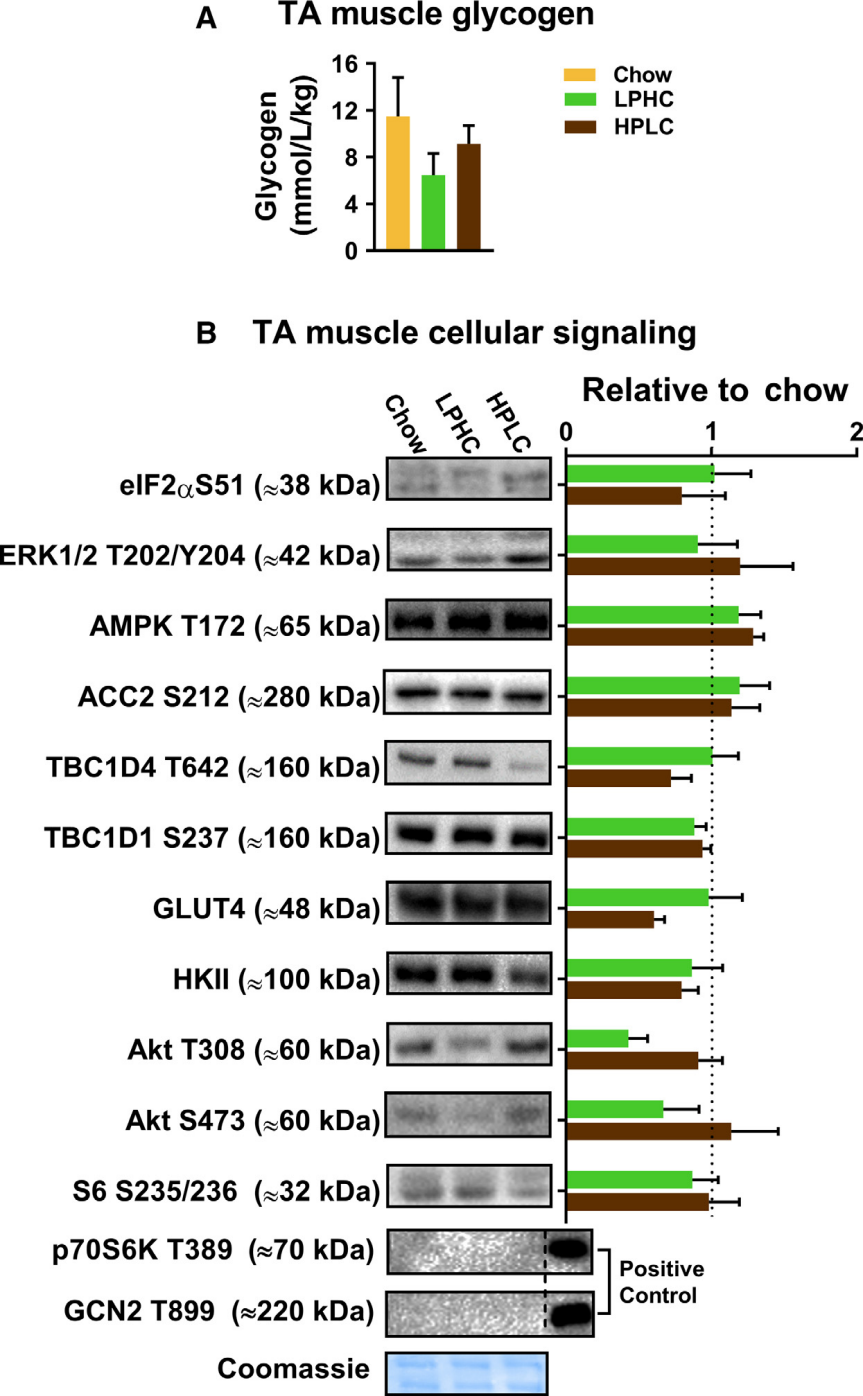
Neither low protein high carbohydrate diet (LPHC) nor high protein low carbohydrate (HPLC) diet altered neither skeletal muscle glycogen content nor cellular signaling. (A) Tibialis anterior (TA) muscle glycogen content. The color legend in panel A is shared with panel B. (B) TA muscle cellular signaling determined by western blotting. The signal intensities are presented relative to chow (dashed line). Representative blots and a coomassie loading control are shown to the left. For p70S6K T389 and GCN2 T899, 25 *μ*g of total protein were loaded, and the positive controls were HEK 293 and 100 nmol/L Calyculin A‐treated HeLa cell lysates, respectively. Data are expressed as mean ± SEM. *N* = 7 for chow and *n* = 8 for LPHC/HPLC.

An FGF21‐adiponectin axis has been proposed to mediate the benefits of low‐protein diet. This would be predicted to activate AMPK in skeletal muscle. Also, our findings in liver of increased glycogen and mTORC1 signaling on LPHC diet prompted us to investigate the same in skeletal muscle, in particular given that the tight correlation between glycogen, AMPK and insulin sensitivity (Wojtaszewski et al. 2002). We therefore also measured glycogen and a number of phosphorylations in TA muscle snap‐frozen immediately after excision. No significant changes were observed in neither glycogen (Fig. 5A) nor cellular signaling (Fig. 5B). Taken together, this suggests that the effects of chronic LPHC and HPLC diets on whole‐body insulin sensitivity are not mediated via skeletal muscle.

## Discussion

Whole‐body insulin sensitivity is inversely associated with the protein content of the diet but the contribution of skeletal muscle, the body′s largest insulin‐stimulated glucose storage site to this association, has not been directly investigated. Presently, we demonstrate that insulin‐sensitivity in isolated skeletal muscle is indistinguishable between diets despite marked changes in whole‐body insulin sensitivity and that cell signaling in skeletal muscle in vivo is not affected despite marked changes in whole‐body insulin sensitivity. This implies that the inverse association between protein content and insulin sensitivity is not mediated via changes in skeletal muscle insulin action.

We presently evaluated insulin action in incubated rodent skeletal muscle, a classical experimental setup to study glucose metabolism (Bonen et al. 1994). The isolated muscle setup relies on simple diffusion of substrates rather than the vasculature, and is limited by glucose transport across the surface membrane rather than intracellular phosphorylation by hexokinase II (Hansen et al. 2000). Therefore, this model isolates the glucose transport step, believed to depend mainly on GLUT4 translocation (Zisman et al. 2000) although this is not entirely clear (Fam et al. 2012). The clear advantage of the incubation model is that it is void of systemic influence and that any phenotype must be a direct and intrinsic feature of skeletal muscle. This model has previously been used to study both insulin sensitizing (Kjobsted et al. 2017) and insulin‐resistance‐promoting conditions (Frosig et al. 2013). However, a limitation of the model is that it does not accurately reflect the in vivo conditions where delivery of glucose and insulin via the vasculature, transport and intracellular metabolism may all regulate the overall rate of skeletal muscle glucose uptake. Therefore, our data, strictly speaking, only rule out an effect of diet on insulin signaling and glucose transport in skeletal muscle. Other sites of regulation might contribute to altered insulin‐stimulated glucose uptake in muscle including muscle blood flow (Sjoberg et al. 2017) and intracellular metabolism (Fueger et al. 2003, 2004). Both adiponectin and BCAA might influence muscle flow via effects on the vasculature (de Boer et al. 2016; Jang et al. 2016). Future studies should ascertain whether low and high‐protein diets might influence skeletal muscle insulin‐stimulated glucose uptake in vivo using, for example, hyperinsulinemic euglycemic clamp studies.

If not skeletal muscle, then what drives the improved glucose metabolism on a LPHC diet? FGF21 appears to be required for the metabolic effects of an LPHC diet (Laeger et al. 2014; Maida et al. 2016). The metabolic improvements in glucose and insulin tolerance induced by recombinant FGF21 required the expression of the FGF21 co‐receptor *β*‐Klotho in white adipose tissue (BonDurant et al. 2017; Li et al. 2018), suggesting a dependence on FGF21‐action in white adipose tissue. Li et al. 2018 suggested that FGF21 promoted healthy expansion of subcutaneous white adipose tissue, increased adiponectin release and anti‐inflammatory M2 macrophage polarization. Adiponectin in turn might influence insulin sensitivity in target organs such as liver and skeletal muscle (Yamauchi et al. 2002; Yano et al. 2008; Vu et al. 2013; Ahlstrom et al. 2017). However, the requirement for adiponectin in mediating the metabolic effects of FGF21 is currently disputed with studies showing either a strong dependency of FGF21 metabolic actions on adiponectin (Holland et al. 2013; Lin et al. 2013) or none (BonDurant et al. 2017). Consistent with a largely adiponectin‐independent mode of action for FGF21, hyperinsulinemic‐euglycemic clamp studies in mice showed that both single‐dose and chronic recombinant FGF21 treatments affected insulin‐sensitivity mainly in liver and adipose tissue but not muscle (Xu et al. 2009a,b). The glycemic improvements by FGF21 in mice are seemingly independent of liver insulin‐sensitization (Emanuelli et al. 2014) but requires functional FGF21 signaling in brown adipose tissue (BonDurant et al. 2017) independent of the major intrascapular brown adipose tissue depot (Emanuelli et al. 2014; Bernardo et al. 2015). Our current data of unaltered in vivo AMPK signaling in skeletal muscle favor that insulin‐sensitization by the LPHC diet is skeletal muscle independent despite the reported increase in adiponectin by the LPHC diet in many studies. Although care should be taken when inferring from the typically higher blood concentration of FGF21 reached in injection studies versus LPHC diet studies, we speculate that the LPHC diet increases brown adipose tissue glucose uptake to improve glycemia similar to what is observed with recombinant FGF21 administration.

Our liver signaling data showed elevated phosphorylation of mTORC1 substrate p70S6K Thr389 on a HPLC diet. However, it is unlikely that mTORC1 hyperactivation suppressed insulin action (Um et al. 2004) in this dietary setting, since a much higher liver phosphorylation of p70S6K in the LPHC diet setting coincided with increased whole‐body insulin sensitivity. No difference in p70S6K signaling was observed in skeletal muscle in response to either diet. It should be noted that although skeletal muscle is believed to play a prominent role in BCAA‐induced insulin resistance, other organs such as adipose tissue and liver might directly or indirectly contribute to BCAA‐metabolism or BCAA‐induced insulin resistance (Herman et al. 2010; Ananieva et al. 2017). Furthermore, not all studies report detrimental effects of elevated BCAA on health parameters (She et al. 2007; D'Antona et al. 2010; Macotela et al. 2011), perhaps because BCAA metabolites rather than BCAAs per se are the driver of insulin resistance. Regardless of the mechanism, our data suggest that skeletal muscle insulin resistance is either not involved in the detrimental effect of HPLC diet on whole‐body insulin sensitivity in normal healthy muscle or that the effect is rapidly washed out ex vivo. The latter seems unlikely if the HPLC diet works mechanistically by promoting lipotoxicity in muscle since high‐fat diet induced insulin resistance in muscle is retained ex vivo (Frosig et al. 2013). However, further studies are needed to clarify this.

In summary, we presently tested whether the changes in insulin sensitivity, induced by a 40 E% high or a 5 E% low‐protein diet, at the whole‐body level correlated with changes in skeletal muscle insulin signaling and glucose transport and found no evidence that skeletal muscle is involved. This suggests that other organs than skeletal muscle are the drivers of the whole‐body changes in insulin sensitivity in these dietary settings.

## Conflict of Interest

All authors declared no competing interests.
